# Comparison of clinicopathological features and long-term prognosis between mixed predominantly differentiated-type and pure differentiated-type early gastric cancer

**DOI:** 10.1186/s12885-021-07962-x

**Published:** 2021-03-06

**Authors:** Yutaka Okagawa, Tetsuya Sumiyoshi, Hitoshi Kondo, Yusuke Tomita, Takeshi Uozumi, Reiichi Iida, Hiroya Sakano, Kaho Tokuchi, Takashi Jin, Masahiro Yoshida, Akira Sakurada, Ryoji Fujii, Takeyoshi Minagawa, Kohtaro Morita, Kei Yane, Hideyuki Ihara, Michiaki Hirayama, Yumiko Oyamada, Shunichi Okushiba

**Affiliations:** 1 Department of Gastroenterology, Tonan Hospital, North 4, West 7, Chuo-ku, Sapporo, Hokkaido 060-0004 Japan; 2 Department of Pathology, Tonan Hospital, Sapporo, Japan; 3 Department of Surgery, Tonan Hospital, Sapporo, Japan

**Keywords:** Gastric cancer, Adenocarcinoma, Histology, Prognosis

## Abstract

**Background:**

Recent studies have shown that mixed predominantly differentiated-type (MD) early gastric cancer (EGC) might have more malignant potential than pure differentiated-type (PD) EGC. However, no study has analyzed all differentiated-type EGC cases treated endoscopically and surgically. This study aimed to compare the differences in clinicopathological features and long-term prognosis between MD- and PD-EGC.

**Methods:**

We evaluated all patients with differentiated-type EGCs who were treated endoscopically and surgically in our hospital between January 2010 and October 2014. The clinicopathological features and long-term prognosis of MD-EGC were compared with those of PD-EGC.

**Results:**

A total of 459 patients with 459 lesions were evaluated in this study; of them, 409 (89.1%) and 50 (10.9%) were classified into the PD and MD groups, respectively. Submucosal invasion was found in 96 (23.5%) patients of the PD group and in 33 (66.0%) patients of the MD group (*p* < 0.01). The rates of positive lymphatic and vascular invasion and ulceration were significantly higher in the MD group than in the PD group (*p* < 0.01). The proportion of patients with lymph node metastasis was also significantly higher in the MD group than in the PD group (5 (10%) vs 6 (1.5%), *p* < 0.01). The 5-year overall and EGC-specific survival rates in the PD group were 88.3 and 99.5%, respectively, while they were 94.0 and 98.0% in the MD group, respectively.

**Conclusions:**

MD-EGC has more malignant potential than PD-EGC. However, the long-term prognosis of MD-EGC is good and is not significantly different from that of PD-EGC when treated appropriately.

## Background

Early gastric cancer (EGC) is defined as a lesion with cancer invasion confined to the mucosa or submucosa, regardless of lymph node metastasis (LNM), according to the Japanese classification of gastric carcinoma (JCGC) [1]. Although endoscopic submucosal dissection (ESD) is well established for EGC treatment and is increasingly performed [2–4], patients with a possible risk of LNM require gastrectomy with lymphadenectomy. The histological type is a crucial determining factor in the treatment strategy for EGC, and the undifferentiated type has been identified as one of the independent risk factors of LNM [5–7]. In Japan, the histological type is determined according to its predominant component [1]; thus, differentiated-type-predominant carcinoma including undifferentiated-type components is classified as differentiated-type carcinoma. In contrast, undifferentiated-type-predominant carcinoma that includes differentiated-type components is classified as undifferentiated-type carcinoma. EGC with mixed histology has been recently reported to possibly have more malignant potential than that with pure histology [8–16]. Previous reports showed that compared with the pure differentiated-type EGC, predominantly differentiated-type EGC including undifferentiated-type components treated with ESD resulted in higher non-curative resection rates [14–16]. Further, predominantly undifferentiated-type EGC with differentiated-type components also had a significantly higher non-curative resection rate than pure-type undifferentiated EGC. Furthermore, EGC with mixed histological type treated with gastrectomy had more LNM than pure histological type as mentioned in previous reports [8–13]. Mixed histological type EGC may have more malignant potential; however, these previous reports have analyzed ESD cases only or gastrectomy cases only, resulting in selection bias. Furthermore, there are only a few reports on the long-term prognosis of mixed histological type EGC patients [17, 18], and these reports also analyzed only endoscopic or surgical cases. To our best knowledge, no study has analyzed all differentiated-type EGC regardless of treatment type (i.e., endoscopic or surgical). Thus, this study aimed to compare the differences in clinicopathologic features and long-term prognosis between mixed predominantly differentiated-type (MD)-EGC and pure differentiated-type (PD)-EGC treated with ESD or gastrectomy and to identify whether MD-EGC can be an appropriate indication of ESD and followed-up without additional treatment after ESD if the criteria of curative endoscopic resection were met.

## Methods

### Study design and patients

This was a single-center, retrospective study conducted at Tonan Hospital, Hokkaido, Japan. The study protocol was approved by the institutional review board of Tonan Hospital, and the study was conducted in compliance with the principle of the Declaration of Helsinki of 1964 and later versions.

We analyzed the medical records, endoscopic reports, and pathological findings of consecutive patients with EGCs who underwent ESD or gastrectomy with lymphadenectomy at our hospital between January 2010 and October of 2014. The inclusion criteria were differentiated-type EGC, age ≥ 20 years, availability of detailed pathological diagnosis, and follow-up more than 5 years or any-cause death within 5 years after the procedures. The exclusion criteria were multiple synchronous gastric cancers, locally recurrent cancers, remnant stomach or gastric tube cancers, and cancers with a neuroendocrine carcinoma component. All resected EGCs were classified by patient age, sex, tumor location, and macroscopic morphological type. The tumor location was divided into the upper, middle, and lower part of the stomach, and the macroscopic type was classified according to the JCGC guidelines [1].

### Histological evaluation

The resected specimens obtained via ESD or gastrectomy were evaluated pathologically according to the JCGC guidelines [1]. When lymphatic invasion was suspected, immunohistochemistry using D2–40 was performed. Vascular invasion was determined using Elastica van Gieson staining. Histological types were classified following the Japanese Gastric Cancer Association (JGCA) guidelines [19]. Well differentiated, moderately differentiated, and papillary adenocarcinoma were classified as differentiated-type carcinoma, while poorly differentiated adenocarcinoma and signet ring cell carcinoma were classified as undifferentiated-type carcinoma. All resected lesions were categorized into either PD or MD types (differentiated-type-predominant carcinoma including undifferentiated-type component) according to the proportions of components at histopathology.

The clinicopathological features analyzed were maximum tumor size, tumor depth, histological type, presence of lymphatic and vascular invasion, ulcerative findings, horizontal margin (for cases treated with ESD), vertical margin (for cases treated with ESD), the rate of endoscopic curative resection (for cases treated with ESD), and the rate of LNM. Additionally, we analyzed the LNM rate of both surgical and ESD cases meeting curative endoscopic resection criteria. The criteria for endoscopic curative resection stipulated in the JGCA guidelines were as follows [19]: differentiated mucosal cancer regardless of size in the absence of ulceration, tumors < 30 mm in diameter in the presence of ulceration, and tumors < 30 mm with invasion into the superficial layer of the submucosa (SM < 500 μm). With respect to MD carcinoma, the lesions with areas of undifferentiated type carcinoma exceeding 2 cm or undifferentiated type component in the part that had invaded the submucosa were defined as non-curative resection. Although the patients with endoscopically non-curative resection underwent additional gastrectomy with lymphadenectomy, some high-risk patients, such as those with advanced age or severe comorbidities, selected non-surgical observation. In curative ESD cases and non-curative ESD cases without additional gastrectomy, LNM negativity was defined as the absence of LNM on follow-up computed tomography (CT) for at least 5 years after ESD.

### Analysis of long-term prognosis

The long-term prognosis was analyzed for the recurrence of gastric cancer, overall survival, and EGC-specific survival at 5 years. In addition, the long-term prognosis of both surgical and ESD cases meeting curative endoscopic resection criteria was analyzed. Survival and recurrence were determined using medical records. The patients who were referred to another hospital after treatment were surveyed by letters to the referred hospitals. The prognosis of patients who completed follow-up were investigated using telephone calls to the patients or their family members.

### Statistical analysis

Quantitative variables were expressed as the mean, while categorical variables were presented as total numbers and percentages. Pearson’s chi-squared test and Mann-Whitney *U*-test were applied as appropriate. Survival rates from the date of treatment were calculated using the Kaplan-Meier method and compared using the log-rank test. A *p*-value  <  0.05 was considered statistically significant. All statistical analyses were performed using EZR (Saitama Medical Center, Jichi Medical University, Saitama, Japan), a graphical user interface for R 2.13.0 (R Foundation for Statistical Computing, Vienna, Austria) [20].

## Results

### Patient characteristics

**Fig. 1:**
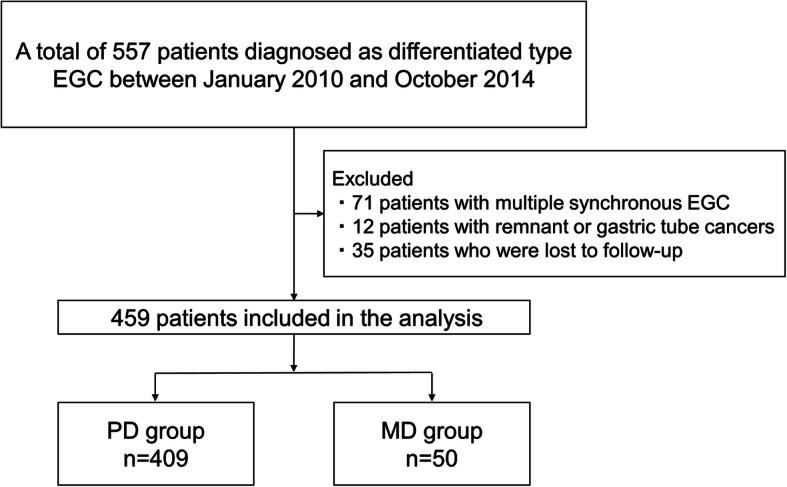
Patient selection flow chart. Abbreviations: EGC: early gastric cancer, PD: pure differentiated-type, MD: mixed differentiated-type

Between January 2010 and October 2014, a total of 681 consecutive patients with 774 EGCs underwent ESD (*n* = 519) or gastrectomy (*n* = 162). Of them, 557 patients with 657 EGCs were diagnosed with differentiated-type EGC. We excluded 98 patients with 198 EGCs due to multiple synchronous gastric cancers (*n* = 71), remnant or gastric tube cancers (*n* = 12), and loss to follow-up within 5 years (*n* = 35). Finally, 459 patients with 459 EGCs were included in the study (Fig. 1). The patient’s characteristics and clinicopathological features are shown in Table 1. There were 351 male and 108 female patients, and the mean patient age was 71.8 years. In total, 103 patients had lesions in the upper third of the stomach; 157 patients, the middle third; and 199 patients, the lower third. Overall, 146 lesions were classified as the elevated type; 4 lesions, flat type; 256 lesions, depressed type; and 53 lesions, complex type. The mean tumor size was 21.9 mm, and 129 (28.1%) lesions were classified as submucosal gastric cancer. Histologically, 409 lesions (89.1%) were classified into the PD group, and 50 lesions (10.9%) were classified into the MD group. The first treatment for EGC was ESD in 396 patients and gastrectomy in 63 patients. The overall rate of non-curative resection for ESD cases was 15.4% (*n* = 61), and 72.1% (*n* = 44) of these cases underwent additional gastrectomy. Overall, 11 patients (2.4%) were found to have LNM: 9 patients based on pathological findings and another 2 patients based on follow-up CT.

**Table 1:** Demographic and clinicopathological characteristics of the patients

All	n (%)
Age (years)	
Mean (range)	71.8 (38–91)
Sex	
Male	351 (76.5)
Female	108 (23.5)
Location	
Upper third	103 (22.4)
Middle third	157 (34.2)
Lower third	199 (43.4)
Macroscopic type	
Elevated (I,IIa)	146 (31.8)
Flat (IIb)	4 (0.9)
Depressed (IIc, III)	256 (55.8)
Complex (elevated + depressed)	53 (11.5)
Tumor size (mm)	
Mean (range)	21.9 (1–128)
Depth	
Mucosa	330 (71.9)
Submucosa, < 500 μm	59 (12.9)
Submucosa, ≥500 μm	70 (15.2)
Histological type	
PD (pure differentiated)	409 (89.1)
MD (mixed predominant differentiated)	50 (10.9)
Lymphatic invasion	35 (7.6)
Vascular invasion	24 (5.2)
Ulceration	66 (14.4)
First treatment	
ESD	396 (84.5)
Gastrectomy	63 (14.7)
Vertical margin + or x (for ESD)	15 (3.8)
Horizonal margin + or x (for ESD)	2 (0.5)
Non-curative resection (for ESD)	61 (15.4)
Additional gastrectomy (for non-curative ESD)	44 (72.1)
LNM	11 (2.4)

Abbreviations: *PD* pure differentiated-type, *MD* mixed differentiated-type, *ESD* endoscopic submucosal dissection, *LNM* lymph node metastasis

### Clinicopathological features

The comparison of clinicopathological findings between PD and MD-EGC is shown in Table 2. There was no significant difference for the mean age and sex between the PD and the MD groups. Regarding the main histological types, the rates of well differentiated carcinoma and moderately differentiated carcinoma were 80.9 and 16.4% in the PD group and 34.0 and 60.0% in the MD group, respectively. There was a significant difference in the main component of carcinoma between the two groups (*p* < 0.01), whereas the locations and macroscopic types of EGC were not significantly different. The maximum size was significantly larger in the MD group than in the PD group (37.3 mm vs 20.1 mm, *p* < 0.01). With respect to the tumor depth, submucosal invasion was found in 23.5% of lesions in the PD group and in 66.0% of lesions in the MD group. EGC with deeper invasion was significantly more frequent in the MD group (*p* < 0.01). Similarly, the rates of lymphovascular invasion and presence of ulcerative findings were significantly higher in the MD group than in the PD group (lymphatic invasion: 28.0% vs 5.1%, *p* < 0.01, vascular invasion: 18.0% vs 3.7%, *p* < 0.01, ulceration: 26.0% vs 13.0%, *p* < 0.01). A total of 90.2% of patients in the PD group and 54.0% of patients in the MD group were treated with ESD, and the difference was statistically significant (*p* < 0.01). The rate of ESD resulting in non-curative resection was significantly higher in the MD group than in the PD group (55.6% vs 12.5%, *p* < 0.01). The rate of LNM was also significantly higher in the MD group than in the PD group (10% vs 1.5%, *p* < 0.01). When we analyzed the LNM rate of both surgical and ESD cases meeting curative endoscopic resection criteria, there was no case with LNM among the curative endoscopic resection cases in both the PD and MD groups (Table 3). In the non-curative cases, the LNM rates were 7.9 and 15.2% in the PD and MD groups, respectively, with no significant difference (*p* = 0.250).

**Table 2:** Comparison of clinicopathological features between the PD and the MD groups

	PD group	MD group	*p*-value
n	409 (%)	50 (%)	
Age (years)			
Mean ± SD (range)	72.1 ± 8.6 (45–91)	69.7 ± 10.0 (38–86)	0.136
Sex			
Male	313 (76.5%)	38 (76.0%)	0.934
Female	96 (23.5%)	12 (24.0%)	
Main component			
Well differentiated	331 (80.9)	17 (34.0)	< 0.01
Moderately differentiated	67 (16.4)	30 (60.0)	
Papillary	11 (2.7)	3 (6.0)	
Location			
Upper third	89 (21.8)	14 (28.0)	0.251
Middle third	139 (34.0)	18 (36.0)	
Lower third	181 (44.2)	18 (36.0)	
Macroscpic type			
Elevated (I,IIa)	131 (32.0)	15 (30.0)	0.388
Flat (IIb)	3 (0.7)	1 (2.0)	
Depressed (IIc, III)	232 (56.7)	24 (48.0)	
Complex (elevated+depressed)	43 (10.5)	10 (20.0)	
Tumor size (mm)			
Mean ± SD (range)	20.1 ± 15.1 (1–99)	37.3 ± 25.8 (4–128)	< 0.01
Depth			
Mucosa	313 (76.5)	17 (34.0)	< 0.01
Submucosa, < 500 μm	48 (11.7)	11 (22.0)	
Submucosa, ≥500 μm	48 (11.7)	22 (44.0)	
Lymphatic invasion	21 (5.1)	14 (28.0)	< 0.01
Vascular invasion	15 (3.7)	9 (18.0)	< 0.01
Ulceration	53 (13.0)	13 (26.0)	< 0.01
First treatment			
ESD	369 (90.2)	27 (54.0)	< 0.01
Gastrectomy	40 (9.8)	23 (46.0)	
Non-curative resection (for ESD)	46 (12.5)	15 (55.6)	< 0.01
LNM	6 (1.5)	5 (10.0)	< 0.01

Abbreviations: *PD* pure differentiated-type, *MD* mixed differentiated-type, *SD* standard deviation, *ESD* endoscopic submucosal dissection, *LNM* lymph node metastasis

**Table 3:** LNM rate of patients with curative and non-curative endoscopic resection in the PD and MD groups

	PD		MD	
n	409 (%)		50 (%)	
	Curative	Non-curative	Curative	Non-curative
	333 (81.4)	76 (18.6)	17 (34.0)	33 (66.0)
LNM	0 (0.0)	6 (7.9)	0 (0.0)	5 (15.2)

Abbreviations: *PD* pure differentiated-type, *MD* mixed differentiated-type, *LNM* lymph node metastasis, *Curative* curative endoscopic resection, *Non-curative* non-curative endoscopic resection

### Long-term prognosis

**Fig. 2:**
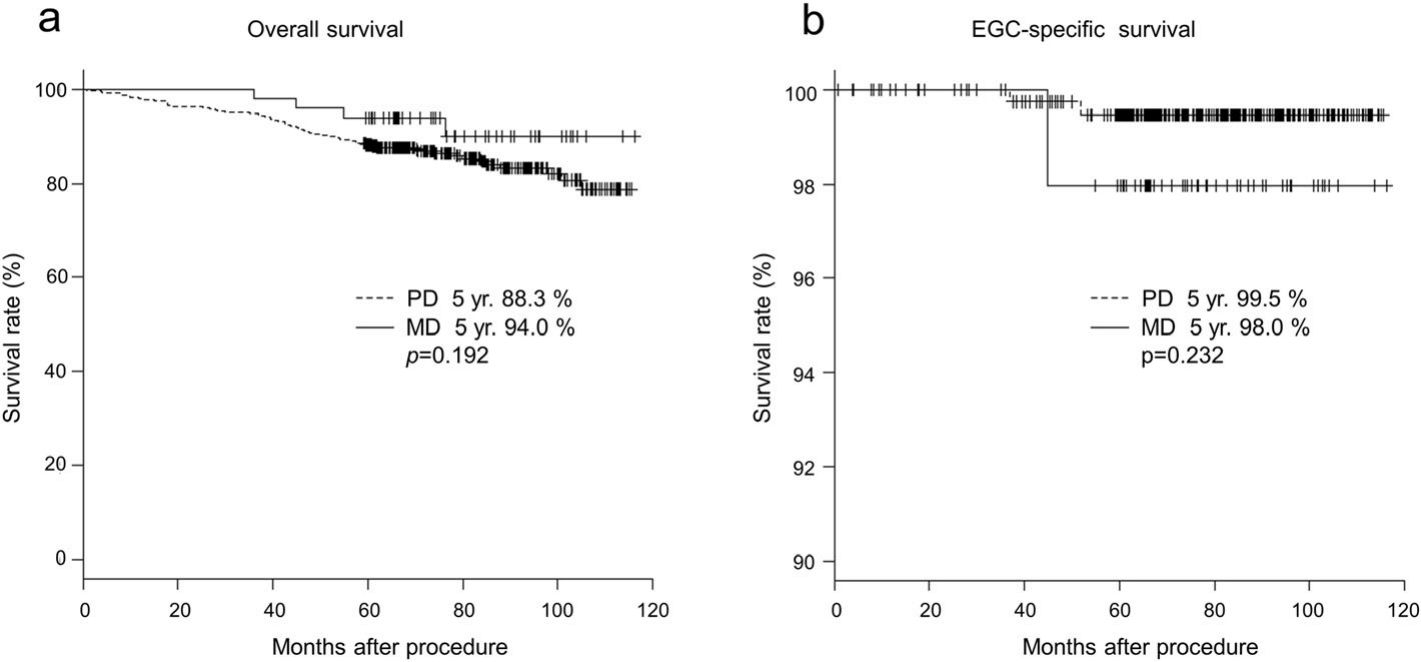
Patient survival rate. **a** 5-year overall survival rate and **b** 5-year EGC-specific survival rate. Abbreviations: EGC: early gastric cancer, PD: pure differentiated-type, MD: mixed differentiated-type

**Fig. 3:**
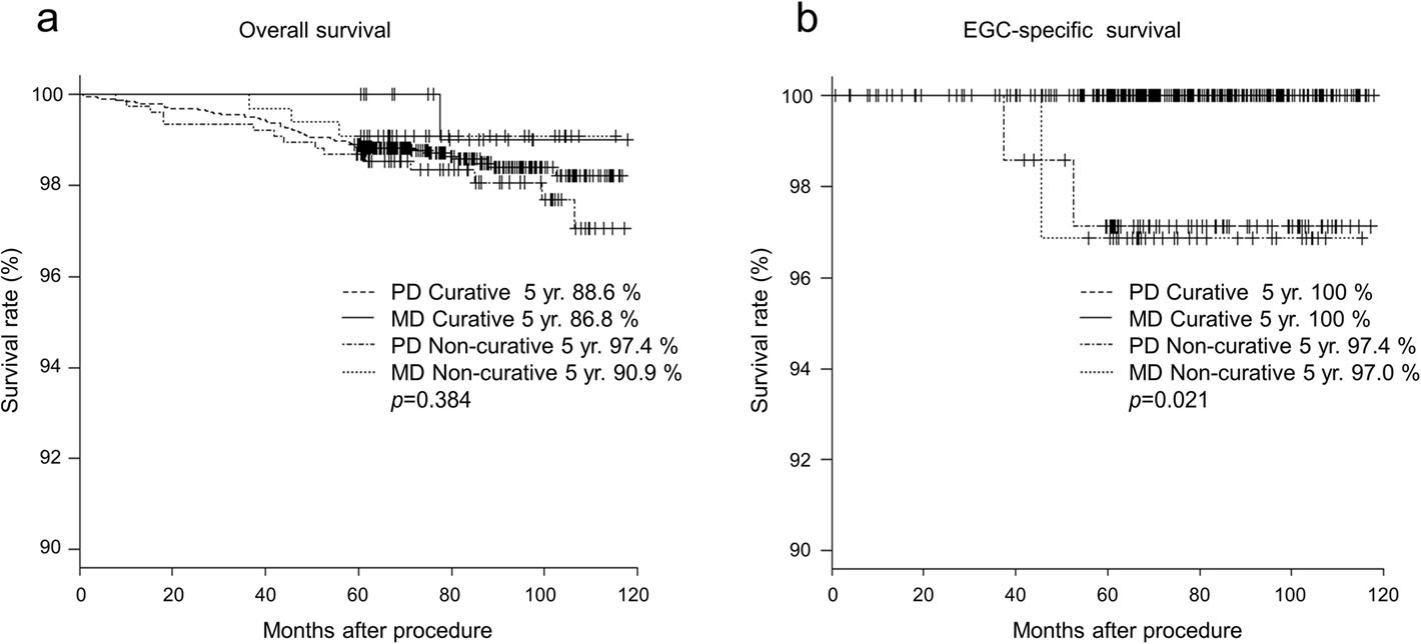
Survival rate of patients with curative and non-curative endoscopic resection. **a** 5-year overall survival rate and **b** 5-year EGC-specific survival rate. Abbreviations: EGC: early gastric cancer, PD: pure differentiated-type, MD: mixed differentiated-type, Curative: curative endoscopic resection, Non-curative: non-curative endoscopic resection

The difference in long-term prognosis between the PD-EGC and the MD-EGC is shown in Fig. 2. The median follow-up period was 71.4 months (range, 0.8–115.7 months) in the PD group and 74.7 months (range, 35.9–116.3 months) in the MD group. There were two and one patients in the PD and MD groups, respectively, who developed EGC recurrence. The two patients in the PD group relapsed at 21.2 and 44.3 months, while the one patient in the MD group relapsed at 29.7 months. All three patients died due to the recurrence of EGC (Table 4). The 5-year overall survival rates in the PD and the MD groups were 88.3 and 94.0%, respectively, with no significant difference (*p* = 0.192). The 5-year EGC-specific survival rates were 99.5% in the PD group and 98.0% in the MD group, and there was also no significant difference (*p* = 0.232). When both surgical and ESD cases meeting curative endoscopic resection criteria were analyzed, the 5-year overall survival rates in the curative PD, curative MD, non-curative PD, and non-curative MD groups were 88.6, 86.8, 97.4, and 90.9%, respectively, with no significant difference (*p* = 0.384). With respect to the 5-year EGC-specific survival rates, they were 100% in both the PD and MD curative groups. However, the 5-year EGC-specific survival rates of non-curative PD and MD groups were 97.4 and 97.0%, respectively, and there were significant differences between the curative and non-curative groups (*p* = 0.021) (Fig. 3).

**Table 4:** Characteristics of the patients with EGC recurrence

Case	Age (years)	Sex	Location	Macroscopic type	Ulceration	Depth	Tumor size (mm)	Histology	LVI	LNM	Treatment	Time to recurrence (months)	Location of recurrence	Outcome
1	75	M	Lower	Ila	present	sm2	12	tub1 > tub2 > pap	present	absent	ESD and Gastrectomy	22.1	Liver, LN	Dead
2	80	M	Upper	I	absent	sm2	30	pap > tub1	absent	absent	ESD	44.3	Liver, LN	Dead
3	73	M	Upper	lla	absent	sm1	20	pap > por2	present	present	Gastrectomy	29.7	Liver	Dead

Abbrevistions: *EGC* early gastric cancer, *LVI* lymphovascular invasion, *LNM* lymph node metastasis, *sm1* submucosa, < 500 μm, *sm2* submucosa, ≥500 μm, *tub1* well differentiated adenocarcinoma, *tub2* moderately differentiated adenocarcinoma, *pap* papillary adenocarcinoma, *ESD* endoscopic submucosal dissection

## Discussion

EGC has good prognosis when treated appropriately. The 5-year EGC-specific survival rate after surgical resection has been reported to be as high as 99% in patients with mucosal cancer and 96% in those with submucosal cancer [21]. However, several recent reports have indicated a difference in malignant potential between PD-EGC and MD-EGC [8–16]. Further, with respect to the long-term prognosis, mixed-type EGC cases with metastatic recurrence following an expanded curative endoscopic resection have been reported [22–24]. In this study, we analyzed all differentiated-type EGC cases endoscopically and surgically resected in our hospital to reduce selection bias. We found no significant difference in mean age and sex between the PD and the MD groups. Although there was also no significant difference in the location and macroscopic types of the lesions between the two groups, the complex type tended to be more common in the MD group. Further, the main component of histological types was significantly different between the two groups. Specifically, compared with the PD group, there were more cases of moderately differentiated carcinoma as the main component in the MD group, consistent with previous findings [9, 14, 17]. The tumor size was significantly larger and the depth was significantly deeper in the MD group than in the PD group. Further, the rates of ulceration, lymphovascular invasion, and LNM were significantly higher in the MD group than in the PD group. These results indicate that MD-EGC might have greater malignant potential than PD-EGC when we analyzed all differentiate-type EGCs treated endoscopically and surgically. With regard to the first treatment of EGC in this study, there was a significant difference in the selection of gastrectomy or ESD between the two groups. We considered that this could be because there were possibly more lesions that were likely to result in endoscopic non-curative resection, such as lesions with submucosal invasion or measuring > 30 mm with ulcer findings, at the time of endoscopic observation in the MD group.

With respect to the long-term prognosis, that were good results in both groups. Only three cases of EGC recurrence were found, and there was no significant difference in the recurrence rate between the two groups. Interestingly, the recurrent cases did not have a single histological type and were all EGC with papillary adenocarcinoma component. According to previous reports [25, 26], EGC with papillary adenocarcinoma component is associated with a higher risk of lymphatic invasion and LNM. Although there was no case of metastatic recurrence in the previous report [26] and we did not assess the histological type that metastasized in our cases, our results may indicate that more careful attention should be paid to the recurrence of EGC with papillary adenocarcinoma component. The 5-year EGC-specific survival rates were 99.5% in the PD group and 98.0% in the MD group, consistent with those in a previous report [21]. The percentage of additional gastrectomy for non-curative ESD cases was relatively high, particularly in the MD group (67.4% in the PD group and 86.7% in the MD group, *p* = 0.152), and those cases were treated based on the current Japanese guidelines [19]. We found that differentiated-type EGC had good prognosis when treated appropriately. In contrast, the 5-year EGC-specific survival rates of cases with non-curative endoscopic resection criteria were worse than those with curative endoscopic resection criteria, regardless of the pure or mixed histological type. These results suggest that there is no major concern for recurrence of mixed histological cases with curative endoscopic resection criteria, but careful follow-up is necessary for non-curative cases even with pure histology. The results of this study support the current Japanese guidelines that define mixed predominantly differentiated-type carcinoma as differentiated-type carcinoma. The 5-year overall survival rate of the PD group was lower than that of the MD group, although there was no significant difference. These results indicate more causes of death other than gastric cancer in the PD group than in the MD group; this may be because the PD group was older than the MD group, although there was no significant difference in age between the groups (the PD group: 72.1 y v.s. the MD group: 69.7 y, *p* = 0.136). Furthermore, the 5-year overall survival rate of the curative group was lower than that of the non-curative group. These differences also may be because the curative group was older than the non-curative group, although there was no significant difference (the curative group: 72.2 y v.s. the non-curative group: 70.6 y, *p* = 0.142).

The present study has several limitations. First, this was a single-center, retrospective study with a relatively small patient size, particularly in the MD group. Further, the number of non-curative resection cases was small, which might have influenced the survival rate. Second, LNM in cases treated with ESD who had not undergone additional gastrectomy could not be evaluated pathologically. Thus, we performed regular follow-up CT for at least 5 years after ESD to evaluate LNM. Third, the rates of non-curative ESD cases without additional gastrectomy were different between the PD and MD groups, and this could have influenced the long-term outcome. Fourth,35 patients (7.1%) were lost to follow-up within 5 years, and their recurrence and survival rates were unknown. Furthermore, because this was conducted in a single center, the possibility of bias in the accumulation of cases treated endoscopically or surgically could not be eliminated. Despite these limitations, this study, which analyzed all differentiated-type EGC cases treated endoscopically and surgically, would be meaningful for considering the biological characteristics of MD-EGC.

## Conclusions

In conclusion, MD-EGC has more malignant potential than PD-EGC. The treatment based on the current JGCA guidelines is important for non-curative ESD cases, particularly those with MD-EGC, which has a high rate of LNM. However, we found that when treated appropriately, the long-term prognosis of MD-EGC is good and is not significantly different from that of PD-EGC. Multicenter trials with a larger number of cases are needed to confirm the results of this study.

## Data Availability

All data generated or analyzed during this study are included in this published article.

## Abbreviations

EGC: Early gastric cancer
LNM: Lymph node metastasis
JCGC: the Japanese classification of gastric carcinoma
ESD: Endoscopic submucosal dissection
MD: Mixed predominantly differentiated-type
PD: Pure differentiated-type
JGCA: the Japanese Gastric Cancer Association
CT: Computed tomography
